# Gut microbiota and meat quality

**DOI:** 10.3389/fmicb.2022.951726

**Published:** 2022-08-23

**Authors:** Binlong Chen, Diyan Li, Dong Leng, Hua Kui, Xue Bai, Tao Wang

**Affiliations:** ^1^College of Animal Science, Xichang University, Xichang, China; ^2^Antibiotics Research and Re-evaluation Key Laboratory of Sichuan Province, Sichuan Industrial Institute of Antibiotics, School of Pharmacy, Chengdu University, Chengdu, China; ^3^Institute of Animal Genetics and Breeding, College of Animal Science and Technology, Sichuan Agricultural University, Chengdu, China

**Keywords:** meat quality, livestock, poultry, antioxidant capacity, fatty acid composition, gut microbiota

## Abstract

Sustainable meat production is important to providing safe and quality protein sources for humans worldwide. Intensive artificial selection and high energy input into the diet of many commercial animals for the last decade has significantly increased the daily gain of body weight and shortened the raising period, but unexpectedly decreased the meat quality. The gastrointestinal tract of animals harbors a diverse and complex microbial community that plays a vital role in the digestion and absorption of nutrients, immune system development, pathogen exclusion, and meat quality. Fatty acid composition and oxidative stress in adipose and muscle tissue influences meat quality in livestock and poultry. Recent studies showed that nutraceuticals are receiving increased attention, which could alter the intestinal microbiota and regulate the fat deposition and immunity of hosts to improve their meat quality. Understanding the microbiota composition, the functions of key bacteria, and the host-microbiota interaction is crucial for the development of knowledge-based strategies to improve both animal meat quality and host health. This paper reviews the microorganisms that affect the meat quality of livestock and poultry. A greater understanding of microbial changes that accompany beneficial dietary changes will lead to novel strategies to improve livestock and poultry meat product quality.

## Introduction

To consumers, meat quality has always been important, especially in the twenty first century (Joo et al., 2013). With the rapid development of animal husbandry, issues relating to the animal’s microbiome have been raised, including the low feed conversion efficiency, nitrogen utilization efficiency, meat quality, and high methane emissions (Nkrumah et al., 2006; Kumar et al., 2013; Gharechahi et al., 2021). To produce high quality meat, it is necessary to understand the characteristics of meat quality traits and the factors that control them.

Meat quality is difficult to define as it is a complex concept that is largely determined by consumer preferences. In a narrower aspect, meat quality is defined as sensory quality, which is the stimulation of the meat product to human senses such as vision, smell, taste, and touch. The sensory quality of meat is evaluated by its color, flavor, pH, drip loss, marbling, tenderness, and juiciness (Thorslund et al., 2016). In a broader sense, meat quality also includes processing quality, nutritional value, and meat hygiene. Fresh meat, as animal tissue that is suitable for use as food, has characteristic qualities that are influenced by various factors (Joo et al., 2013) such as muscle structure, chemical composition (i.e., fatty acid composition, intramuscular fat, and carbohydrate content), chemical environment, interaction of chemical constituents, postmortem (p.m.) changes in muscle tissues, stress (such as oxidative stress), pre-slaughter effects, product handling, processing and storage, and microbiological numbers and populations. For example, the fatty acid composition can easily be altered though feeding especially in monogastric animals. Meanwhile, carbohydrate content is more closely related to genetics in animals such as pigs; feeding in the last days before slaughter and handling at slaughter (both ante- and postmortem) (Aaslyng and Meinert, 2017). Other factors also play important roles in meat quality, such as exposure to other organisms including *Campylobacter* and *Salmonella*, which play an important role in the quality and safety of end-products from these animals. Additional steps, including washing and chilling of the meat within the production cycle, aim to control the proliferation of these microbes as well as those which cause product spoilage (Marmion et al., 2021). Storing meat under different temperatures (Kaur et al., 2021; Liang et al., 2021) also aims to control these microbes. In addition, microbiota also play crucial role during meat fermentation (Ferrocino et al., 2018). Here, we will focus on the meat quality of fresh meat.

The composition and relative proportions of dominant gut microbial groups vary animal among species (Richards et al., 2005). The gut microbiota is believed to influence many metabolic processes such as (van Kuijk et al., 2021) nutrient absorption (Xu et al., 2013), host health, and the meat quality (Richards et al., 2005). By comparing the skeletal muscle of germ-free mice to the pathogen-free mice, germ-free mouse skeletal muscle showed atrophy, decreased expression of insulin-like growth factor 1 of host mice (Lahiri et al., 2019). In ducks, the abundance of genus *Prevotella*, *Lactobacillus*, and *Lachnospiraceae UCG-008* was significantly lower in meat production ducks (Qin et al., 2019). Saccharolytic and anaerobic microbiota can especially aid in the degradation of host-indigestible carbohydrates (such as cellulose and resistant polysaccharides) into monomeric or dimeric sugars, and subsequently ferment them into short-chain fatty acids (SCFAs) (Tremaroli and Bäckhed, 2012; Koh et al., 2016), which can be carried by the host’s systemic circulation to reach extraintestinal organs and make broad-range impacts on the host (Silva et al., 2020; Gautier et al., 2021). Among the SCFAs, butyrate is the preferred energy source for colonocytes and has been investigated most extensively, other absorbed SCFAs drain into the portal vein (Koh et al., 2016). For example, acetate can reduce appetite *via* a central homeostatic mechanism by crossing the blood-brain barrier (Frost et al., 2014). Acetate-dependent GPR43 stimulation in the white adipose tissue improved glucose and lipid metabolism (Kimura et al., 2013). High-fiber diet (produce high amounts of propionate) protect against allergic airway through inducing hematopoiesis of dendritic cells that seed the lungs (Trompette et al., 2014).

**FIGURE 1:**
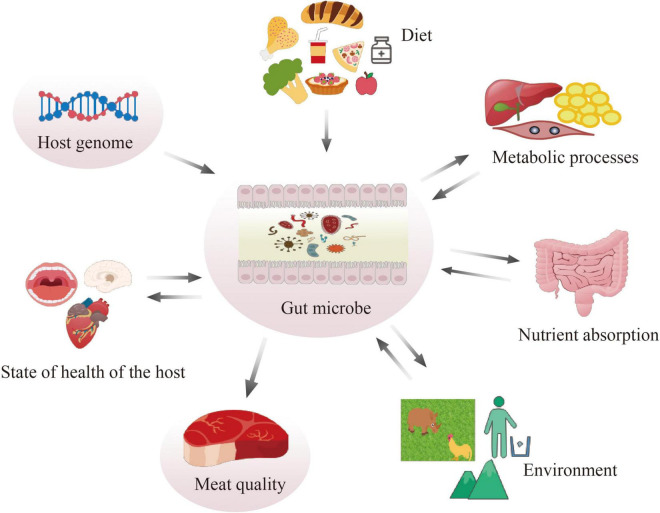
Function of gut microbiota.

There is a growing interest in understanding the role of the gut microbiome on meat quality related traits (Muinos-Buhl et al., 2018). Genetic background, in addition to diet composition, might impact gut microbiota composition (Lopez-Garcia et al., 2021; Figure 1). For carcass composition and meat quality traits in pigs, heredity of the microbiome was estimated. High positive microbial correlation was found among different traits, particularly with traits related to meat color and firmness score (Khanal et al., 2021). Better understanding of microbial composition could aid the improvement of complex traits (Khanal et al., 2021). There were also differences in the diversity and composition of the microbial community among swine breeds; among these breeds, Duroc, known for its excellent meat quality, tenderness, improved flavor, and palatability, showed different microbial community from other breeds (Alain et al., 2014; Pajarillo et al., 2015). Symbiotic supplementation into the diet improved the growth performance, oxidative stability, and meat quality in both chicken (Cheng et al., 2017) and duck (Chen et al., 2018). Furthermore, the gut microbiome is also well-acknowledged as a key element in regulating fat deposition: since they are closely related to meat quality, excessive lipid accumulation and high oxidative stress have become a serious health and economic problem in the pig industry (Zhao et al., 2022).

This paper reviews the microorganisms affecting the meat quality in livestock and poultry. A better understanding of the livestock and poultry gut function and microbiology will provide us new opportunities for the improvement of meat quality in animal production.

## Gut microbiota affect intramuscular fat deposition

Animal fat deposition is a complex biological process. Obesity has been shown to be highly related to gut microbial profile (Portune et al., 2017), as gut microbiota imbalance contributes to lipid deposition (Kallus and Brandt, 2012; Zhao, 2013). Since many of the bacterial taxa correlated to the intramuscular and subcutaneous fat depots did not overlap, the gut microbiota likely impacted adipose accumulation largely *via* separate adipogenic pathways (Krause et al., 2020). Abundance in the genera of unclassified *Erysipelotrichaceae* and *Butyrivibrio* increased in pigs fed with rice distillers’ by-products and induced an improvement in animal growth and fat deposition (Nguyen Cong et al., 2019). Fat deposited in muscle includes intermuscular fat and intramuscular fat (IMF). As IMF is a key factor affecting meat qualities such as tenderness, juiciness, and taste (Hausman et al., 2009), it is an economically important factor in animal breeding (Fang et al., 2017). Excluding age, sex, and nutrition, gut microbiome and genetics are two important factors affecting IMF (Zheng et al., 2022). As a newly discovered factor of IMF, the gut microbiome is reported in recent years in various animals (Lahiri et al., 2019; Qi et al., 2019; Wen et al., 2019; Chen et al., 2021; Liu et al., 2021a).

**FIGURE 2:**
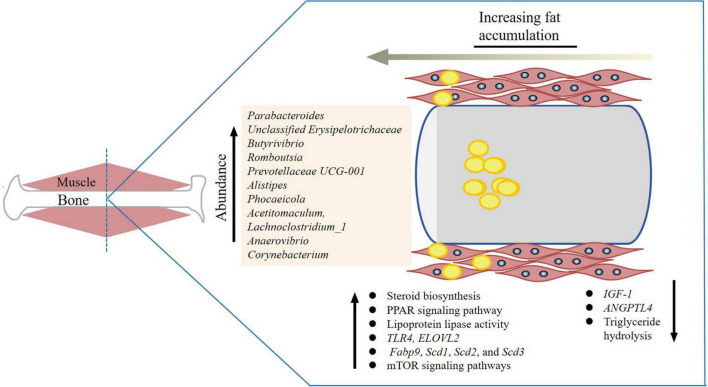
Gut microorganisms related to intramuscular fat accumulation. The yellow dots are lipid droplets and the bacterial genera related to intramuscular fat (IMF) content are listed. The genes and pathways are also listed.

The gut microbiome was found to play a crucial role in varying tendencies for fattiness in different breeds of pigs (Lei et al., 2021). The Duroc breed had higher IMF content than Landrace and Large White (Bergamaschi et al., 2020). Gut microbiome studies showed that the higher relative abundances of the genera *Ruminococcaceae_NK4A214_group*, *Parabacteroides*, *Christensenellaaceae_R-7_group*, and *Ruminiclostridium* might corelate with higher IMF content (Lei et al., 2021; Figure 2). In addition, differences of colonic bacterial abundances and bacterial metabolites between fatty- and lean-type pigs were detected (Jiang et al., 2016). Similarly, other studies showed that an elevated ratio of *Firmicutes* to *Bacteroidetes* and increased abundance of genus *Romboutsia* in colonic samples was correlated with higher IMF content in pigs (Wu et al., 2021). Obese Jinhua pigs had better meat quality that is associated with higher IMF content than lean Landrace pigs. To show that this trait was related to gut microbiota, mice were given microbiota from each pig species. The mice receiving Jinhua pig’s microbiota had elevated lipid and triglyceride levels and the lipoprotein lipase activity, as well as reduced *ANGPTL4* expression in the muscle (Wu et al., 2021). This increase was also accompanied with an elevated ratio of *Firmicutes*/*Bacteroidetes* and increased abundance of *Romboutsia*. *Prevotella copri*, and could increases fat accumulation by activating host chronic inflammatory responses through the *TLR4* and mTOR signaling pathways. This also significantly upregulated the expression of the genes related to lipogenesis and fat accumulation (*Fabp9*, *Scd1*, *Scd2*, and *Scd3*) (Chen et al., 2021). In Enshi pigs, the microbiota genera *Prevotellaceae UCG-001* and *Alistipes* in the cecum and *Clostridium sensustricto 1* in the jejunum were shown to be highly, positively correlated with IMF (Tang et al., 2020). IMF content in the longissimus muscle was increased with antibiotic exposure, which increased expression of genes related to fatty acid uptake and *de novo* synthesis and decreased expression of genes related to triglyceride hydrolysis (Yan et al., 2020).

In ruminants, a previous study found that anti-*Porphyromonas gingivalis* antibody titers were positively correlated with intramuscular adipose tissue content (Watanabe et al., 2021). Castration in male cattle increased adiposity via small intestinal microbial alterations (Whon et al., 2021), which resulted in a greater increase in the subcutaneous and intramuscular fat, and a higher meat tenderness score than those of non-castrated bulls (Field, 1971; Marti et al., 2017). The castrated male cattle harbored distinct ileal microbiota dominated by the family *Peptostreptococcaceae* (Whon et al., 2021). Angus beef meat, which is superior in juiciness, tenderness, and flavor due to a higher intramuscular fat (IMF) content, was found to have a significantly higher relative abundance of *Roseburia*, *Prevotella*, and *Coprococcus* Angus (Zheng et al., 2022). The gut microbial species *B. uniformis*, *R. inulinivorans*, *B. vulgatus*, *C. catus*, *E. rectale*, and *F. prausnitzii* were all found to be positively correlated with the muscular metabolism-related genes including *MSTN*, *ATP2A1*, *MYLPF*, *ACTN3*, *MYL1*, and *TNNT3* and have positive effect on meat quality in cattle (Zheng et al., 2022). The mucin-degrading bacterium *Akkermansia*, known for regulating energy expenditure, was enriched in Brahman calves that contained less IMF content, while butyrate-producing bacterium *Faecalibacterium* was linearly positively correlated with Angus, which is known for its high IMF (Fan et al., 2019). The phyla *Tenericutes* and *Saccharibacteria* (formerly known as TM7) were negatively correlated in longissimus lipid content of Angus steers (Krause et al., 2020). Increased dietary energy improved beef C18:1 cis-9, C18:2n-6 trans, monounsaturated fatty acids (MUFAs) and IMF content, and decreased C18:0, C18:1 trans, C22:0, C20:3n-3, C22:6n-3, and saturated fatty acids (SFAs) in Holstein bulls (Wang H. et al., 2019). These changes were accompanied by an increase in abundance of the genera *Prevotellaceae_UCG-004*, *Phocaeicola*, *Acetitomaculum*, *Lachnoclostridium_1*, *Prevotellaceae_UCG-003*, and *Anaerovibrio*.

In chickens, *Methanobrevibacter* and *Mucispirillum schaedleri* were identified to be significantly correlated with fat deposition: chickens with a lower *Methanobrevibacter* and a higher *M. schaedleri* abundance had significantly lower abdominal fat (Wen et al., 2019). The *ELOVL2* gene, which is involved in long-chain polyunsaturated fatty acid elongation and lipid synthesis (Jakobsson et al., 2006; Gregory et al., 2013; Pauter et al., 2014), was found to be associated with feed utilization in correlation with a higher abundance of *Corynebacterium* (Wen et al., 2021). The bacteria related genes and pathways affecting IMF content that are discussed here are summarized in Figure 2.

## Bacterial metabolites short-chain fatty acids improved meat quality traits

Fatty acids are carboxylic acids with an aliphatic chain. The varying fatty acid contents and profiles of adipose tissues and muscles influence the meat characteristic qualities in pigs, sheep, and cattle (Enser et al., 1996; Wood et al., 2008). Fatty acid composition defines the firmness/oiliness of adipose tissue and muscle oxidative stability, which in turn affects flavor and muscle color (Wood et al., 2008). Meat is considered as a major source of polyunsaturated fatty acids (PUFA), which are essential for humans, creating a concerted effort to increase the production of PUFAs in livestock.

Microbiota residing in the livestock and poultry gastrointestinal tract digest and ferment food consumed by animals into nutrients which are utilized by the host to produce meat and milk (Liu et al., 2021c). Among the metabolites produced by the beneficious gut microbiota, short-chain fatty acids (SCFAs) have received increasing attention because of their important role in disease prevention and recovery (Sanderson, 2004). SCFAs are part of a microbial fermentation product in the gut which may be related to the reduced body fat of rabbits. Acetate, a major SCFA in rabbit gut, reduced intramuscular triglyceride levels via increasing fatty acid uptake and fatty acid oxidation. *PPAR*α was also found to be associated with the acetate-reduced intracellular fat content (Liu et al., 2019). SCFAs levels, especially butyrate level, had critical impacts on finishing weight of rabbit. Gut microbiome explained nearly 11% of the variation in finishing weight (Hu et al., 2021). Low fat content pigs had higher abundances of butyrate-producing bacteria species that improved the formation of SCFAs, especially butyrate, thus alleviating fat deposition, while high fat-content pigs had a higher abundance of *Archaeal* species along with higher methanogenesis functions, leading to more efficient fat deposition (Zhao et al., 2022). SCFA administration into the ileum could improve the meat quality of growing pigs by inhibiting the mRNA expressions of fatty acid synthase (FAS) and acetyl-CoA carboxylase in the longissimus dorsi (Jiao et al., 2021).

In addition, SCFAs have been demonstrated to play important roles in maintaining morphology of the small intestine wall (Mannelli et al., 2019) in addition to offering energy to host cells as well as gut microflora (Jiao et al., 2021). As they are commensal fermentation products, the levels and compositions of SCFAs are influenced by dietary fiber intake and consumption of SCFA-enriched foods (Yamamura et al., 2020). Especially in these ruminants, rumen microbes ferment feed, and produce volatile fatty acids (VFAs), the main energy source for the host (Miura et al., 2021). Stable rumen fermentation, with an increase in *Prevotella* spp. and unclassified *Bacteroidales* in fattening periods, has been shown to be one of the most important factors to producing high-quality meat in cattle (Miura et al., 2021). In goats, the contents of saturated fatty acids (SFAs) C14:0, C16:0, and C18:0 were significantly higher in the alfalfa (*Medicago sativa* L.) fed group. The microbe *Prevotella_1* was negatively correlated with C18:0 and positively correlated with C16:1, while *Clostridium* and *Romboutsia* showed a positive correlation with monounsaturated fatty acids (MUFAs) and PUFAs (Wang Y. et al., 2022).

**FIGURE 3:**
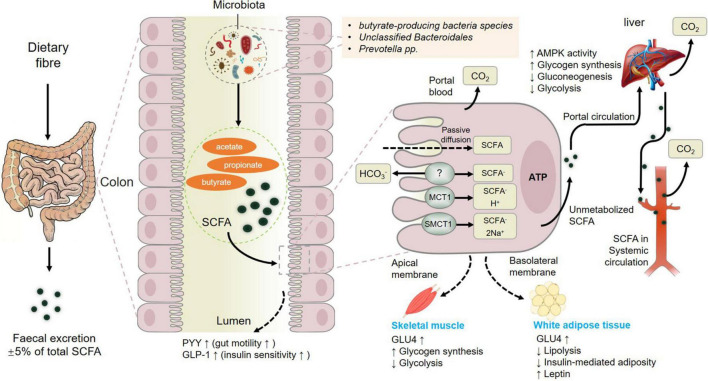
Metabolism of short-chain fatty acids (SCFAs) from dietary fiber to systemic circulation. MCT1, monocarboxylate transporter 1; SMCT1, sodium-dependent monocarboxylate transporter 1; GLP-1, glucagon-like peptide-1; GLUT-4, activated glucose trans-porter protein-4; PYY, peptide YY. Figure based on the study of Dalile et al. (2019).

In summary, fermentation of dietary fiber by commensal gut bacteria in the colon leads to the production of short-chain fatty acids (SCFAs) including acetate, propionate and butyrate (Liu et al., 2021d), which are rapidly absorbed by colonic cells *via* monocarboxylate transporters, passive diffusion, or bicarbonate (HCO3^–^) exchange *via* an unidentified exchange mechanism (Figure 3). The SCFAs including acetate, butyrate, and propionate are converted to acetyl-CoA or propynyl-CoA by pathways involving the acetyl-CoA carboxylase (ACSSs) and beta oxidation. This step will produce ATP, which contributes to the maintenance of cell homeostasis. SCFAs that are not metabolized by colonic cells travel *via* the basolateral membrane into the portal circulation to the liver, providing an energy substrate for hepatocytes *via* oxidation (Dalile et al., 2019). Thus, only small amounts of the SCFAs produced in the colon reach systemic circulation. The colon derived SCFAs that do manage to reach systemic circulation promote anti-inflammatory and immunomodulatory effects as well as increasing insulin secretion, maintaining energy homeostasis and improving the function of the gut, liver, skeletal muscles, and adipose (Figure 3). These results showcased the positive effects of SCFAs from gut microbiota on muscle (Liu et al., 2021a) and fat tissue to further influence meat quality.

## Diet improves meat quality by altering gut microbiota

Food and nutraceuticals are important for the gastrointestinal microbiome composition of humans and other animals (Lanng et al., 2021). Poor-quality food items consumed as main meals were reported to relate to severe irritable bowel syndrome in humans, as an enrichment of gut microbiota with a function toward a specific type of hydrogen metabolism associated with animal carbohydrate metabolism were detected in these cases (Tap et al., 2021). The dietary glucose oxidase could inhibit harmful bacteria and promote beneficial bacteria, which could be related to the improvement of the growth performance and intestinal barrier function in chickens (Zhao et al., 2021). *Eubacterium rectale* subspecies harboring flagellin-encoding genes were associated with a predominantly meat-based diet (Tap et al., 2021). Two of the three *E. rectale* subspecies were found to be associated with lower gut microbial community diversity, higher host BMI, and higher fasting blood insulin levels (Costea et al., 2017). Higher protein intake during resistance training does not enhance strength, but modulates gut microbiota in middle-aged adults (McKenna et al., 2021). Intramuscular fat, fibrosis, and the number of pro-inflammatory cells in rats after high-fat high-sugar feeding increased by 3-days and was sustained across 28-days, compared to control-diet animals (Collins et al., 2016). Nutrients have a great influence on the state of the body through gut microbiota community, and this effect on meat animals is to affect their meat performance.

In these monogastric mammals, such as pigs fed on high-fructose corn syrup increased subcutaneous fat and triacyl glycerides in plasma compared to sucrose fed pigs, but IMF did not differ between diets (Maj et al., 2021). Most of the IMF-associated operational taxonomic units (OTUs) in pigs belonged to the bacteria genera related to polysaccharide degradation and amino acid metabolism, such as *Prevotella*, *Treponema*, *Bacteroides*, and *Clostridium* (Fang et al., 2017). Pigs that received fermented complete feed (FCF) had better meat quality, which was indicated by a higher unsaturated fatty acid content and a lower average back-fat thickness. This feed also significantly reduced the relative abundances of presumably pathogenic bacteria of genus *Escherichia–Shigella* (Tang et al., 2021). *L. plantarum* ZJ316 was found to have probiotic effects, which could improve the meat quality of pigs, including chewiness, gumminess, and restoring force, by inhibiting of the growth of opportunistic pathogens (Suo et al., 2012).

In poultry, the effects dietary fiber on cecal SCFA concentrations and cecal microbiota of chickens showed that there were interactions between bird breed and dietary fiber, based on the concentrations acetic acid and total SCFA (Walugembe et al., 2015). In caged chickens, adding lignocellulose increased the microbial diversity and the abundance of the butyrate-producing bacteria *Faecalibacterium* and *Roseburia* (Hou et al., 2020), which could in turn reduce the production of proinflammatory factors. The addition of Yingshan Yunwu green tea polysaccharide conjugates increased chicken breast muscle pH and redness-greenness (a*) value and increased the abundance of *Bacteroidetes* and *Lactobacillus* and decreased the abundance of *Proteobacteria* (Xiang et al., 2020).

Especially in ruminants, vitamin E is an essential nutrient that stabilizes PUFA and has a central role in meat quality. Extra dietary vitamin E has been shown to have a beneficial effect on the growth performance, oxidative stress biomarkers, carcass characteristics, and meat quality of lambs (Maraba et al., 2018). Meat quality was improved in lambs that had a high fiber and low protein, which might corelated with *Planctomycetaceae* (OTU1882) abundance (Akonyani et al., 2021). Alterations in the gut microbiota with a high-rice (HR) diet improved the meat quality of goats, which was displayed as a significantly reduced lightness of the meat at 45 min and 24 h after slaughter. The abundance of *Oscillibacter* increased, while *Phocaeicola* and *Christensenellaceae_R-7_group* significantly decreased with the HR diet (Wang K. et al., 2021). Dietary administration of L-carnitine can improve feed efficiency and modulate the ruminal and intestinal microbiota of lambs, ruminal fermentation was also improved with higher concentration of SCFA were detected (Martin et al., 2022).

Thus, healthy and high-quality foods for all animals is necessary. To avoid the generation of food waste products, nutraceuticals should be developed into a sustainable approach that can be implemented in commercial, antibiotic-free livestock and poultry to provide safe and high-quality meats (Tolnai et al., 2021).

## Antioxidant and meat quality

Stress inevitably occurs in the journey from the farm to abattoir in modern livestock husbandry (Xing et al., 2019). An excess of free radicals will trigger oxidative stress (Estévez, 2015), leading to harmful effects on DNA, proteins, and lipids (Xing et al., 2019). Oxidative stress that is characterized as elevated reactive oxygen species (ROS) levels could result in the deterioration of meat quality, degenerative health problems, and could lead to great economic losses for the industry every year (Fellenberg and Speisky, 2006; Xing et al., 2019). Oxidative stress occurs in farmed animals when free radical production exceeds the capacity of the antioxidant defense system. Oxidized diets were one of the most effective factors on oxidative stress in livestock and poultry (Xing et al., 2019). A significant increase in some benign intestinal bacteria (*Lactobacillus* etc.) and a significant decrease in harmful bacteria (*Turicibacter* and *Helicobacter*) was found to diminish oxidative stress in mice (Liu et al., 2021b), which indicated the crucial function of gut microbiota in oxidative stress regulation. In addition, oxidized pork stimulated oxidative stress and inflammation by altering gut microbiota in mice (Ge et al., 2020). Understanding how the gut microbiota contributes to oxidation and how oxidative stress alters the meat quality and protein functionality as well as the sensory, nutritional, and shelf-life quality of meat (Zhang et al., 2013) is important.

Meat becomes susceptible to oxidative processes due to high levels of unsaturated fatty acids (UFAs) and multiple initiators such as transition metal, and certain oxidoreductase enzymes (Lund et al., 2011). Thus, optimization of UFAs composition of meat in livestock and poultry is desirable (Li et al., 2012). *Butyrivibrio proteoclasticus*-related bacteria extensively hydrogenate PUFA to saturated fatty acids (SFA). For example, they are responsible for the conversion of *trans*-vaccenic acid (C18:1) to stearic acid (C18:0) (Li et al., 2012), resulting in the high ratio of SFA/PUFA (Huws et al., 2011). In fish, the proportions of unsaturated to saturated fatty acids as well as *Bacillus* species increased in response to temperature changes (Tsuda et al., 2015). Dietary polyunsaturated fatty acids (PUFAs) were reported to activate hepatic AMP kinase (Suchankova et al., 2005) and PUFAs to induce a partitioning of fatty acids toward oxidation rather than lipogenesis, increasing the production of free radicals (Viollet et al., 2006). Feeding 100% enzymatically digested food waste did not alter the meat quality of pigs compared with pigs fed with traditional diet but contained more omega-3 fatty acids (Jinno et al., 2019). The use of *Lactobacillus johnsonii* as a probioticexhibited a positive effect on muscle lipid peroxidation by significantly increasing superoxide dismutase (SOD) and attenuating the decrease of intramuscular fat, C18:3n-3 (α-linolenic acid, ALA), C20:4n-6, C20:5n-3 (eicosapentaenoic acid, EPA), C22:4n-6, C22:5n-3, C22:6n-3 (docosahexaenoic acid, DHA), total PUFA, and n-3 PUFA (Wang et al., 2017).

In addition, supplementing antioxidants in the diet also has a dual effect for the commercial animals by improving meat quality and maintaining growth performance (Xie et al., 2022). In Japanese quails, essential oil (EO) supplementation was found to be beneficial, and should be recommended for improving the meat quality (Kurekci et al., 2021). Meanwhile, butyrate, in combination with forskolin, alleviates necrotic enteritis, increases feed efficiency, and improves carcass composition of broiler chickens (Yang et al., 2022). In chickens, dietary supplementation with magnolol improved meat quality with a balanced gut microbiota homeostasis of increased *Faecalibacterium* and decreased *Coprobacillus* in the cecum, and increased glutathione (GSH), superoxide dismutase (SOD), and total antioxidant capacity (T-AOC) levels in breast muscle and jejunum (Xie et al., 2022). Protocatechuic acid (PCA) increased the relative abundance of *Firmicutes* and *Actinobacteria* while reducing *Bacteroidetes* and *Proteobacteria*, thus improved the feed efficiency, growth performance, meat quality, and antioxidant capacity of broilers. It also enhanced intestinal immune function and improved the structure of intestinal flora to favor improved intestinal health in Chinese, yellow-feathered broilers (Wang Y. et al., 2019). Dietary supplementation of onion leaf powder in chickens exerted antimicrobial, immunomodulatory, and antioxidant effects (Adeyemi et al., 2021), in addition to showing an increase in cecal *Lactobacillus* spp. counts. Azolla at 10% supplementation affected phase-feeding and increased oxidative stress in chicken (Abdelatty et al., 2021). On the other side, toxins have opposite effect on meat quality compared to the antioxidant. After aflatoxin B1 (AFB1) exposure, mutton quality was impaired, which was reflected by the changed structure of muscle fibers in addition to other changes (Cao et al., 2021). AFB1 caused changes in the levels of oxidative stress indicators T-SOD, T-AOC, MDA, and GSH, as well as changing the GSH/GSSG ratio, and decreasing the abundances of *Butyrivibrio*, all of which are related to the quality of the mutton (Cao et al., 2021).

**FIGURE 4:**
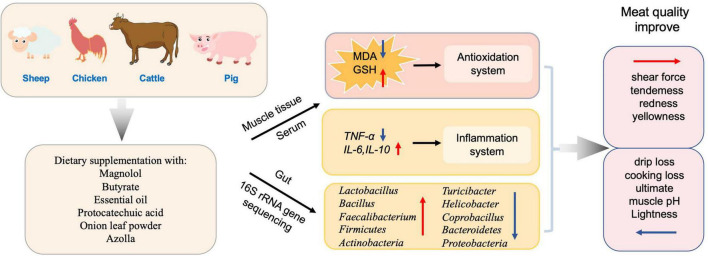
Antioxidants affect the composition of the gut microbiota and meat quality.

In total, these diet supplemented antioxidants (such as magnolol, butyrate, essential oil etc.) could alter gut microbiota composition and decrease oxidative stress and further improve the meat quality of livestock and poultry (Figure 4).

## Different rearing systems and microbiota

As a result of consumer demand for high-quality products and legal livestock and poultry welfare requirements, rearing systems have been the focus of scientific research for many years (Bogosavljevic-Boskovic et al., 2012). The choice of rearing system is a highly important parameter of meat characteristics (Meluzzi et al., 2009), with organic production giving better quality meat. Outdoor access also appears to improve meat characteristics (Ponte et al., 2008). Studies across multiple experimental and commercial systems have shown greater fear and anxiety in the indoor-raised animals (Campbell et al., 2020). Rearing systems alter gut microbiota composition, and can hence influence the immune system (Thoene-Reineke et al., 2014) and meat quality. For example, comparisons of intestinal permeability, morphology, and ileal microbial communities of commercial hens housed in conventional cages and cage-free housing systems are different (Wiersema et al., 2021).

Previous studies have compared housing system effects on microbiota (Yan et al., 2021), such as birds housed in indoor versus outdoor systems (Seidlerova et al., 2020; Schreuder et al., 2021). Free-range hens had richer *Actinobacteria*, *Bacteroidetes*, and *Proteobacteria* (Cui et al., 2017) species diversity. The rearing system causes changes of behavior, microbiome, and gene expression in chickens (Chen et al., 2019). Cage-free environments generated higher gut microbiota diversity (Hubert et al., 2019).

Indoor enrichment (more space, straw bedding) generally improved pig meat juiciness and flavor through higher IMF concentration (Lebret, 2008). Berkshire pigs, known for their high-quality meat and adaptation to semi-grazing conditions (Jang et al., 2018), were shown to have increased fat area in muscle and adipose tissue and the myofiber density significantly in the pigs of the free grazing farm group. The relative abundance of bacteria associated with lipid metabolism (such as *Oscillibacter*) and probiotic function (*Lactobacillus* and *Clostridium*) was also higher (Qi et al., 2019). The Tibetan pig (*Sus scrofa*), which also has been known for its meat quality, showed a higher abundance of *Bacteroides* and *Fibrobacterota* under semi-grazing conditions (Niu et al., 2022).

In ruminants, compared to feedlot-fed beef, grazed beef had lower fatty acid content and higher vitamin content (Prache et al., 2020). Rumen *Bacteriodetes*, involved in carbohydrate and lipid metabolism and lipoic acid metabolism, further broke down fatty acids (Hart et al., 2018). In both dairy and beef cows, grazing was found to affect metabolic properties of gut microbiota (including *Firmicutes*, *Bacteroidetes*, *Proteobacteria*, *TM7*, and *Actinobacteria*) (Kim and Wells, 2016), thus also affecting their skeletal muscle characteristics and compounds of meat (Mwangi et al., 2019). The significant differences in gut microbe composition between grazing and feedlot Angus beef might have an impact on the meat quality of the meat (Zhang et al., 2021). Nellore cattle fed with concentrated diets at adaptation day had a higher content of SCFAs and relative higher abundance of *Succinivibrio* (Pinto et al., 2020). In the muscles of sheep, artificial pasture grazing feeding increased the level of PUFAs in meat, especially the concentrations of n3 PUFA, and reduced n6/n3 (Wang B. et al., 2021). Also in in these artificial pasture grazing sheep, the abundance of ruminal *Moryella* was decreased, *Schwartzia* and *Anaeroplasma* were increased, both of which were strongly correlated with the n3 PUFA in the muscle of lambs. Among the SCFAs, the propionic acid content was higher in the novelty group, where pullets were presented with novel objects such as balls, bottles, bricks, brooms, brushes, buckets and changed at weekly intervals than the control group chickens, which had rice hulls as floor litter only. The indoor and outdoor hens could be differentiated by their gut microbiota profiles (Bari et al., 2021).

Overall, the physical environment (such as indoor/outdoor access, individuals per pen, etc.) is a major criteria that drives the phenotypic development and ultimately impacts animal wellbeing (Zeineldin et al., 2019). Artificial pasture grazing livestock and free-range chickens could alter the gut microbiome composition, increasing abundances of good bacteria genera such as *Lactobacillus*, *Actinobacteria*, *Bacteroidetes*, *Proteobacteria*, and *Clostridium* genus, and hence improve the immune system activity and meat quality of livestock and poultry.

## Zoonotic diseases related microorganisms

When we discuss microbes and meat quality, there is one category we cannot ignore, which has a more important role and far-reaching impact, and that is zoonotic pathogens. It may be bacterial, viral or parasitic, or may involve unconventional agents and can spread to humans through direct contact or through food, water or the environment, resulting in infectious disease that is transmitted between species from animals to humans (or from humans to animals) (Rahman et al., 2020). The “Asia Pacific strategy for emerging diseases: 2010” report estimated that around 60% of the emerging human infections are zoonotic in nature and among these pathogens more than 70% originated from wildlife species (Jones et al., 2008; Li and Kasai, 2011). Domestic animals also play a significant role in the transmission of various diseases to humans and in many cases, they work as amplifiers of pathogens emerging from wild animals (Morand et al., 2014). Cattle, sheep, goats, dogs, cats, horses, pigs, and other domestic animals act as reservoirs of pathogens of domestic zoonoses and can transmit the diseases to humans (Chowdhury et al., 2021). Pathogens can be transmitted through direct contact or animal-origin foods. Of these zoonotic diseases transmitted by domestic animals, anthrax caused by *Bacillus anthracis* poses significant public health importance. *B. anthracis* is soil-borne bacteria with the capability to produce spores; thus, allowing them to survive in the environment for a very long time. Anthrax which can be transmitted to humans through close contact with infected animals (such as cattle and goats) or their products (such as meat, milk, skin, hides, or even bones) (Goel, 2015). Among the bovine zoonoses having serious public health significance, tuberculosis is the most important zoonotic disease. The disease has been a significant cause of severe economic loss in animal production (including meat and milk production). It is caused by *Mycobacterium bovis*, *M. tuberculosis*, or rarely *M. caprae* (Torgerson and Torgerson, 2010; Bayraktar et al., 2011). Brucellosis is one of the most common bacterial zoonotic diseases causing over 500,000 human cases throughout the world every year (Hull and Schumaker, 2018). Among the twelve species of the genus *Brucella*, *Brucella melitensis*, *B. abortus*, *B. suis*, and *B. canis* are zoonotic in nature. The common transmission pattern of brucellosis to human occurs through the consumption of unpasteurized milk or milk products, though the human–human transmission is rare. Meat, dairy products, and eggs are the main ways by which people are exposed to zoonotic bacteria. *S. aureus*, *Salmonella* species, *Campylobacter* species, *Listeria monocytogenes*, and *Escherichia coli* are the major zoonotic bacterial pathogens that are the causative agents of food-borne illness and death in the world associated with the consumption of contaminated animal products (Abebe et al., 2020). More than 90% of bacteria-triggered food-borne illnesses are caused by *Salmonella* spp. and *Campylobacter* spp. (Thorns, 2000). All domestic livestock, including poultry, can act as a reservoir for bacteria causing food-borne illnesses (Sobur et al., 2019; Alam et al., 2020; Ievy et al., 2020). These bacteria may enter the food chain from production of food animals up to the final consumption of animal products. Most human infectious diseases have animal origins. These pathogens do not only cause diseases in animals, but they also pose a serious threat to human health. Therefore, when we pay attention to zoonotic diseases related microorganisms, meat quality is no longer our primary consideration, its impact on health is the most important thing. Robust active surveillance targeting all components of the “One Health” approach needs to be implemented to early and accurately detect zoonoses, so that effective control measures could be taken to protect public health.

## Conclusion

**FIGURE 5:**
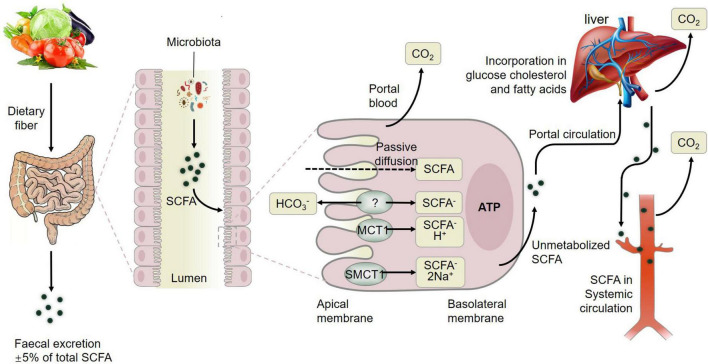
Gut microbiota and meat quality.

The cultivation of sustainable livestock and poultry meat production is crucial to providing safe and quality protein sources for human nutrition worldwide (Shang et al., 2018). Gut microbiotas play important roles in the digestion and absorption of nutrients. The symbiotic interactions between the host and microbe is fundamental to livestock species’ health, productivity, and meat quality-related traits (Yeoman and White, 2014). Here, we reviewed recent studies about the relationship between microbiota and meat quality in livestock and poultry, which showed that some gut microbiotas have a potential role in influencing meat quality, indicating that diet and housing could affect the microbial community, bacterial metabolites, and finally change meat quality (Figure 5). Understanding the role of the gut microbiome on meat quality and production related traits in livestock species will be useful in developing new tools for improving the meat production systems and industry in the future.

## Author contributions

BC, DLe, HK, and XB wrote the manuscript. DLi and TW supervised the work. All authors contributed to the article and approved the submitted version.
